# Supplementary material for: Synthesis of Pyridoxine-Derived Dimethylpyridinols Fused with Aminooxazole, Aminoimidazole, and Aminopyrrole
Source: Molecules. 2022 Mar 23;27(7):2075. doi: 10.3390/molecules27072075 (PMC9000659; doi:10.3390/molecules27072075)
Supplement: Supplementary file 1 [file molecules-27-02075-s001.zip › molecules-1648605-supplementary.pdf]

Supplementary Material

for

Synthesis of pyridoxine-derived dimethylpyridinols fused with aminooxazole, aminoimidazole, and aminopyrrole

Bhuwan Prasad Awasthi, Hyunji Lee and Byeong-Seon Jeong

^{1}H and ^{13}C NMR spectra of all new compounds

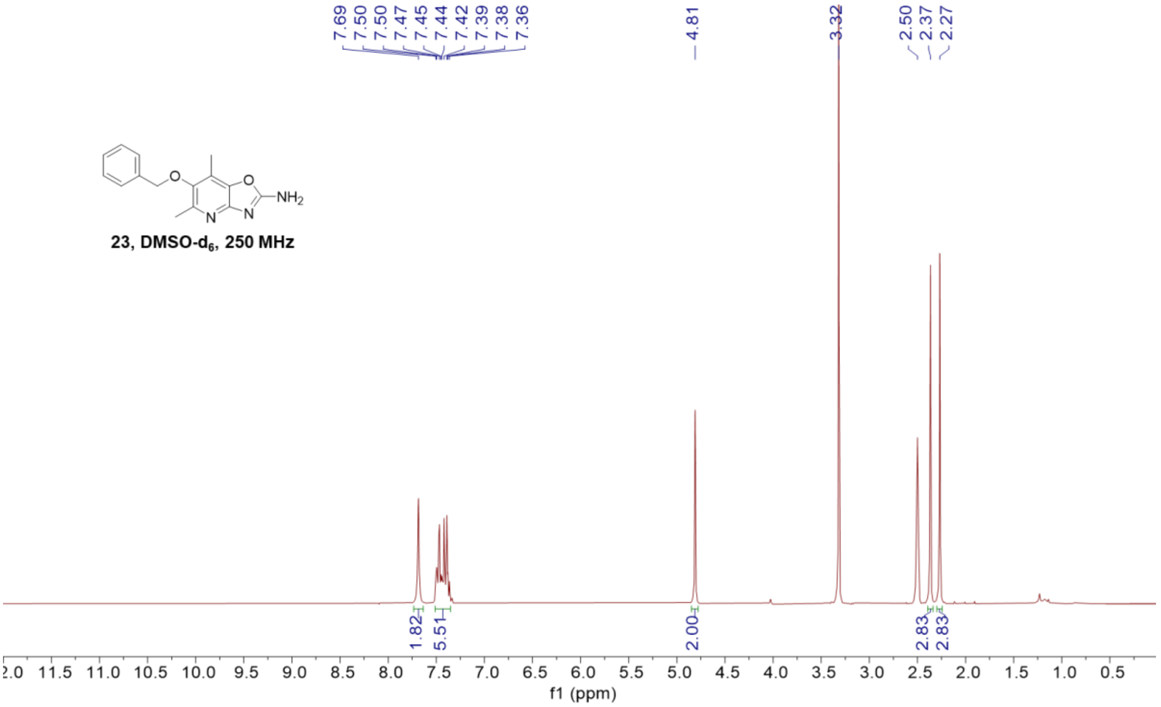

Figure S1. ^{1}H-NMR Spectrum of compound **23**.

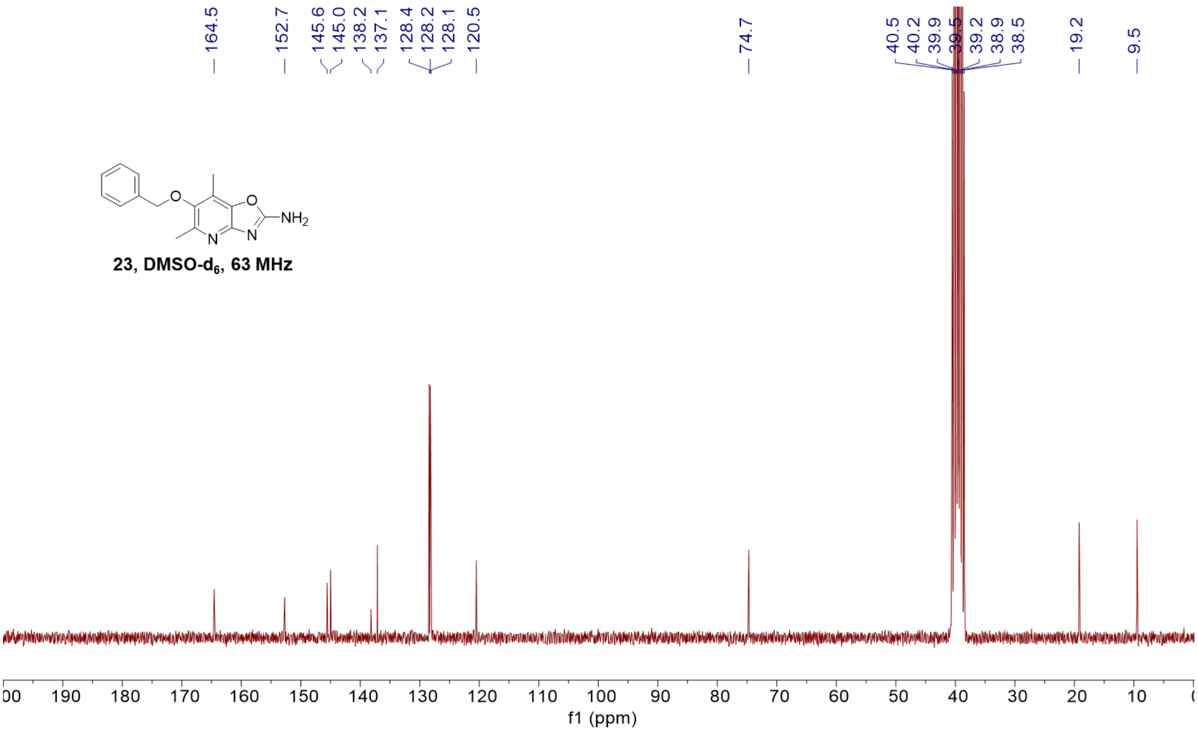

Figure S2. ^{13}C-NMR Spectrum of compound **23**.

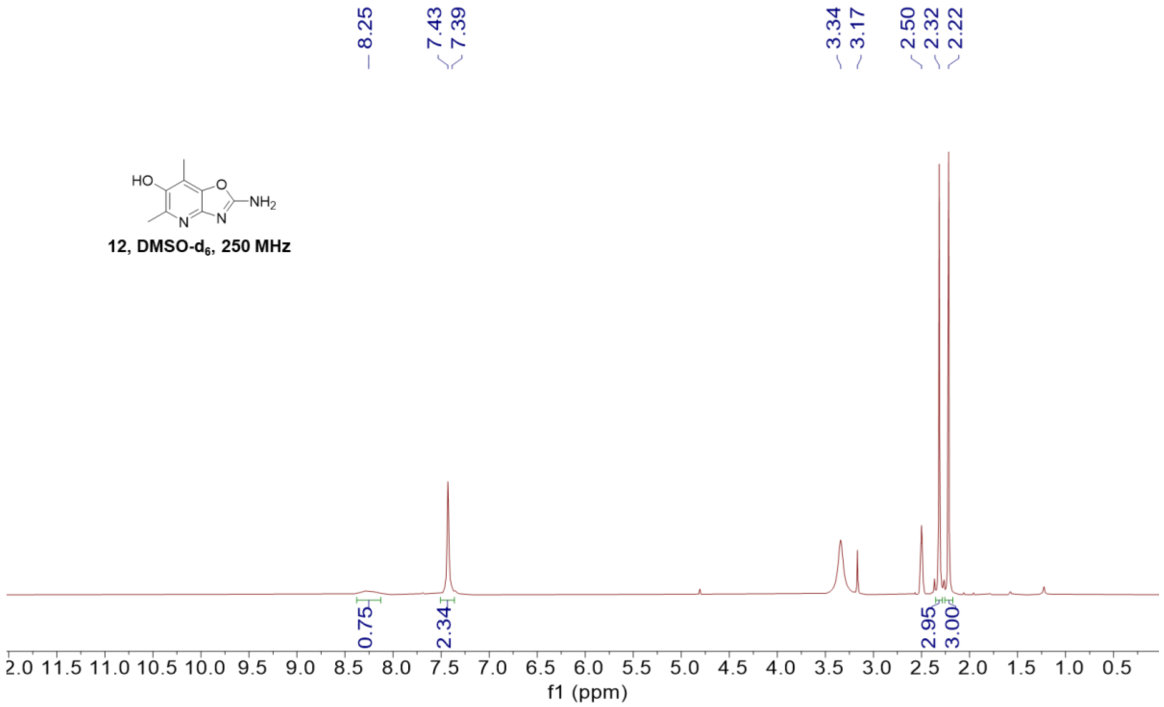

Figure S3. ^{1}H-NMR Spectrum of compound **12**.

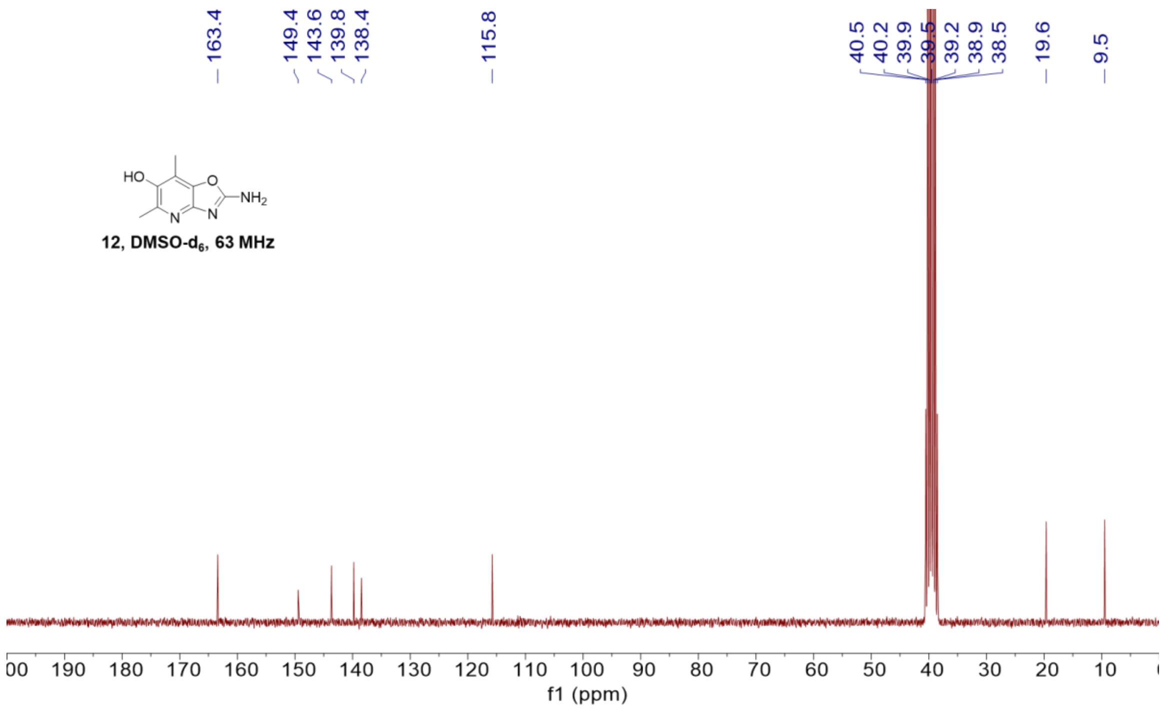

Figure S4. ^{13}C-NMR Spectrum of compound **12**.

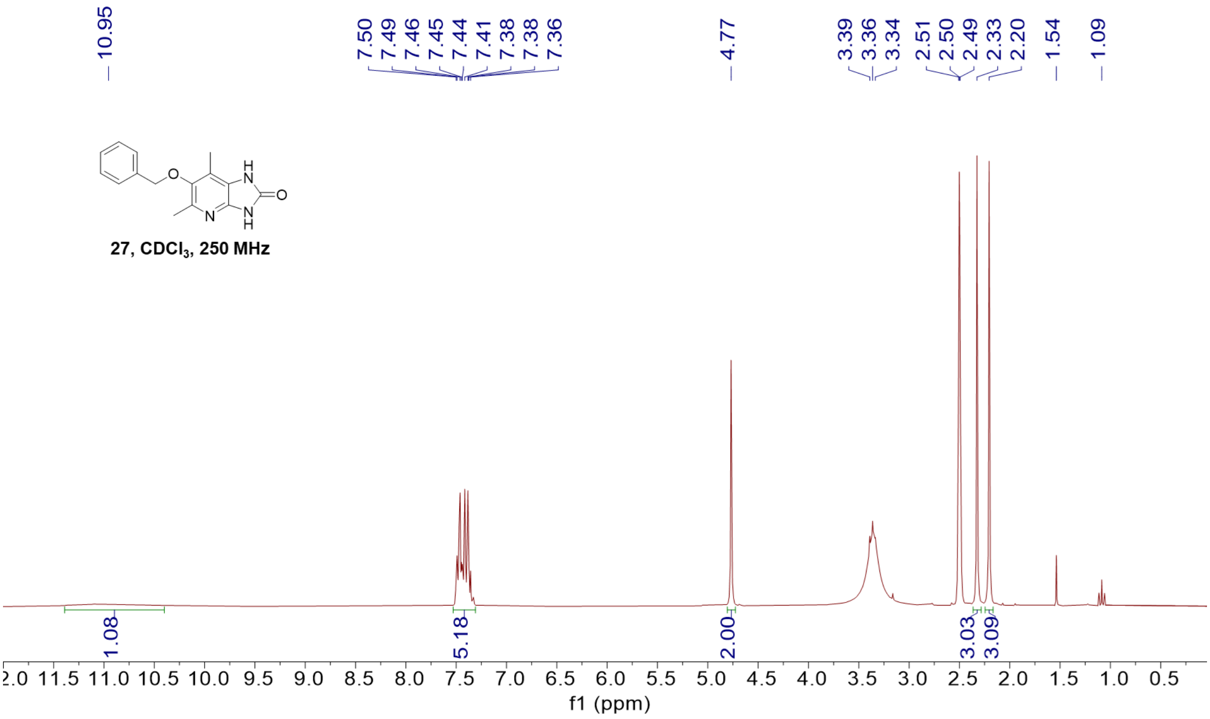

Figure S5. ^{1}H-NMR Spectrum of compound **27**.

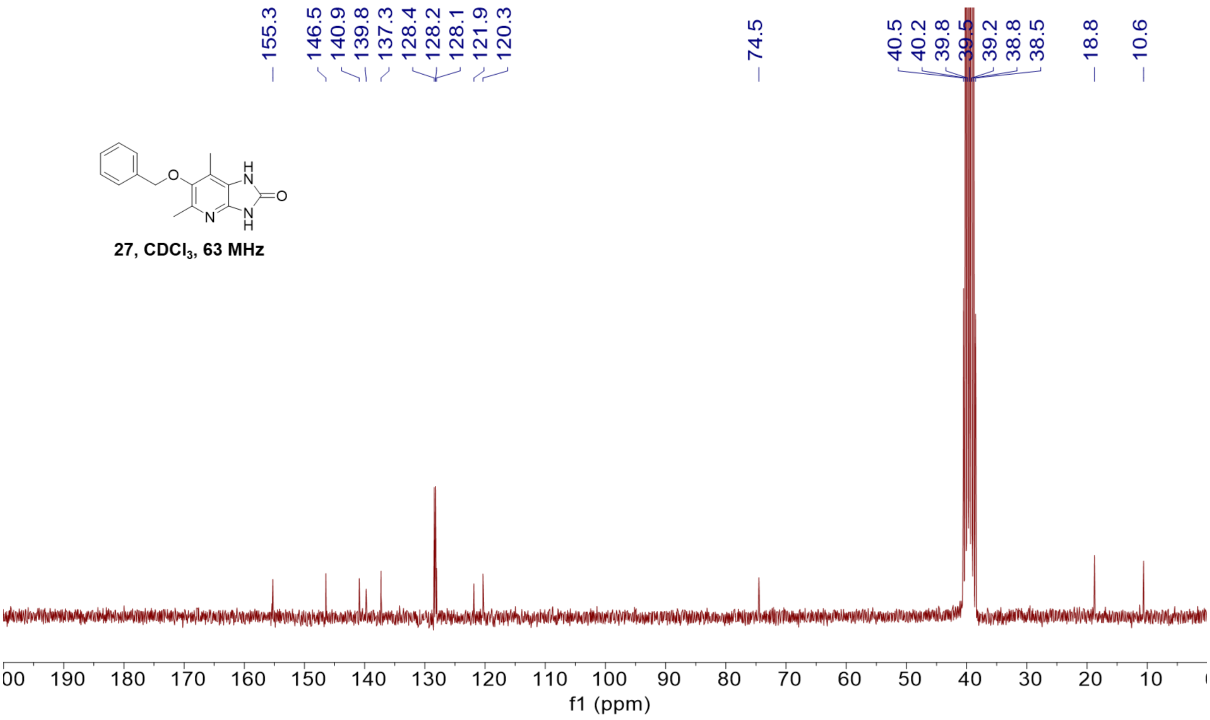

Figure S6. ^{13}C-NMR Spectrum of compound **27**.

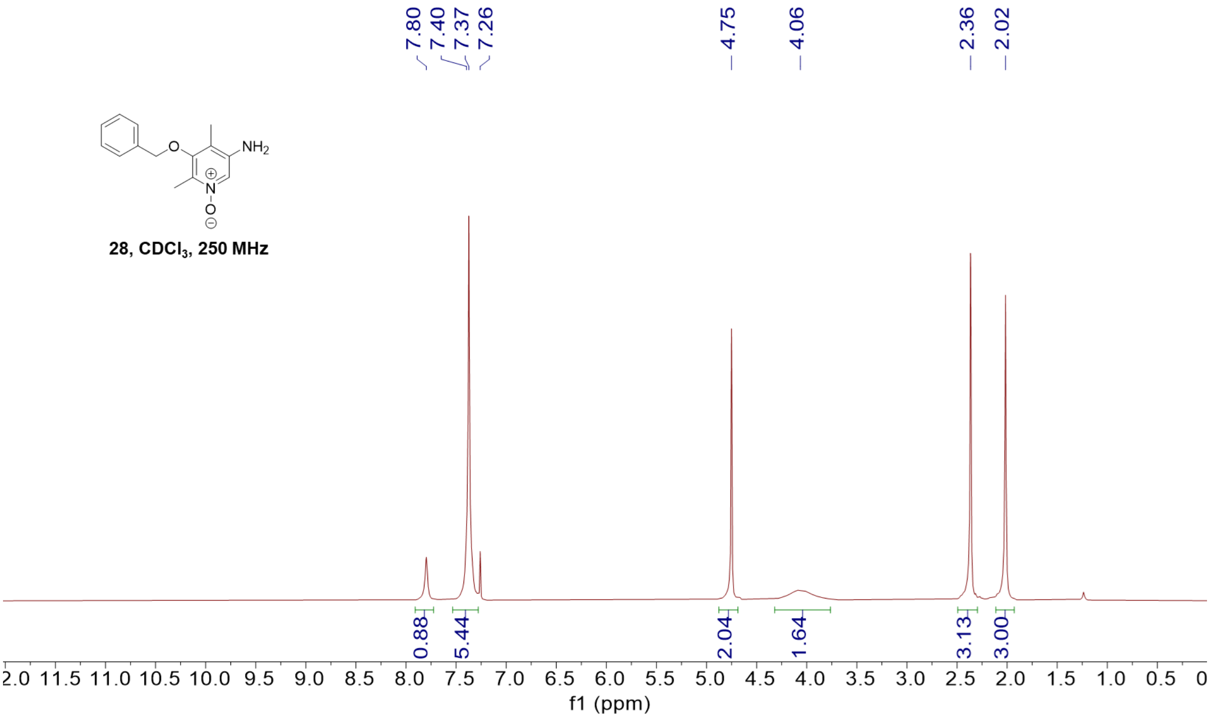

Figure S7. ^{1}H-NMR Spectrum of compound **28**.

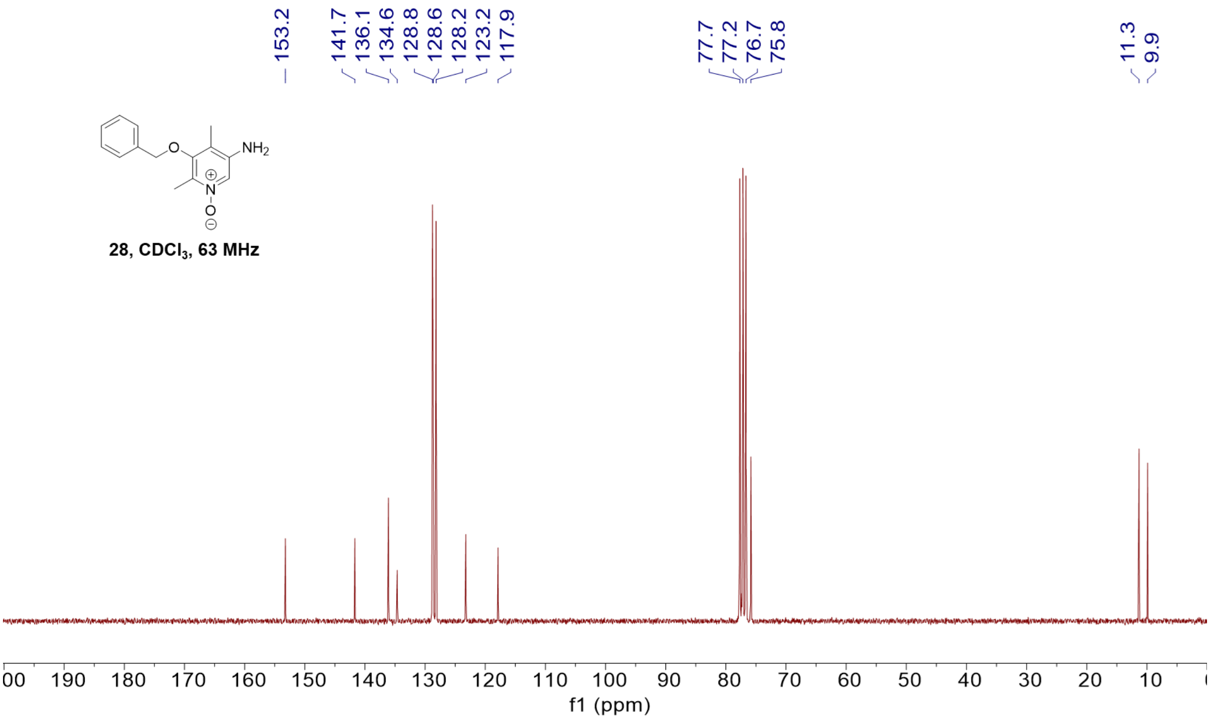

Figure S8. ^{13}C-NMR Spectrum of compound **28**.

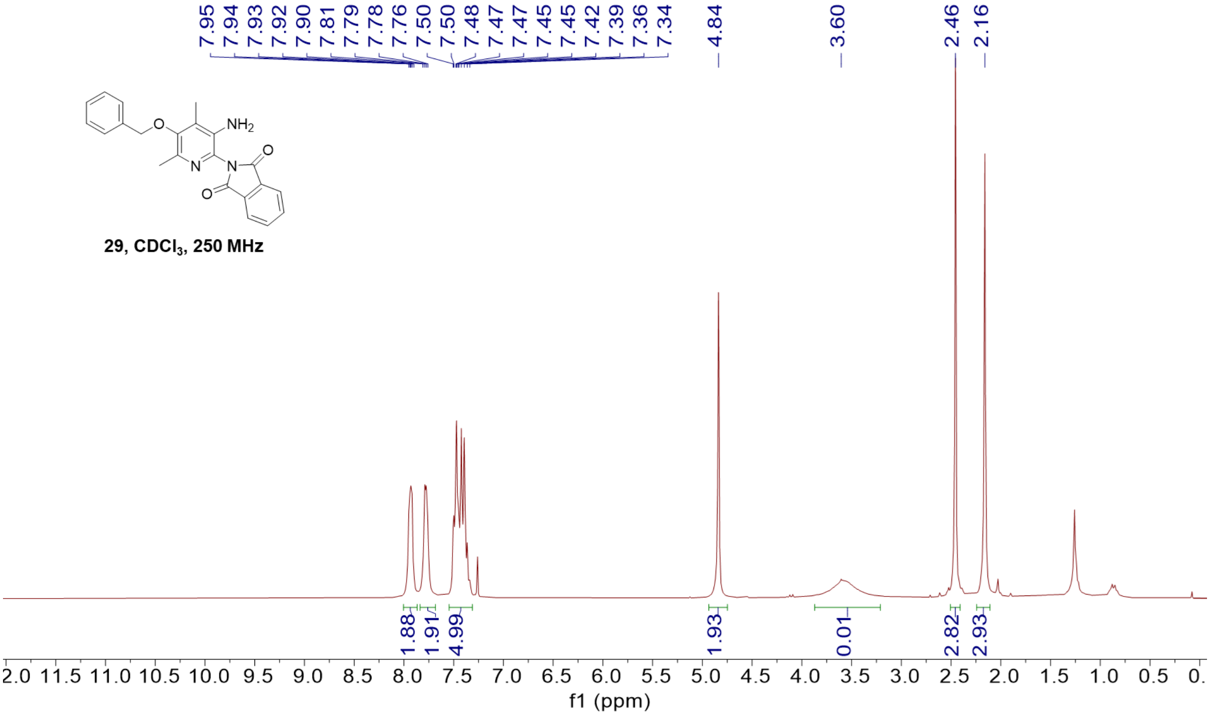

Figure S9. ^{1}H-NMR Spectrum of compound **29**.

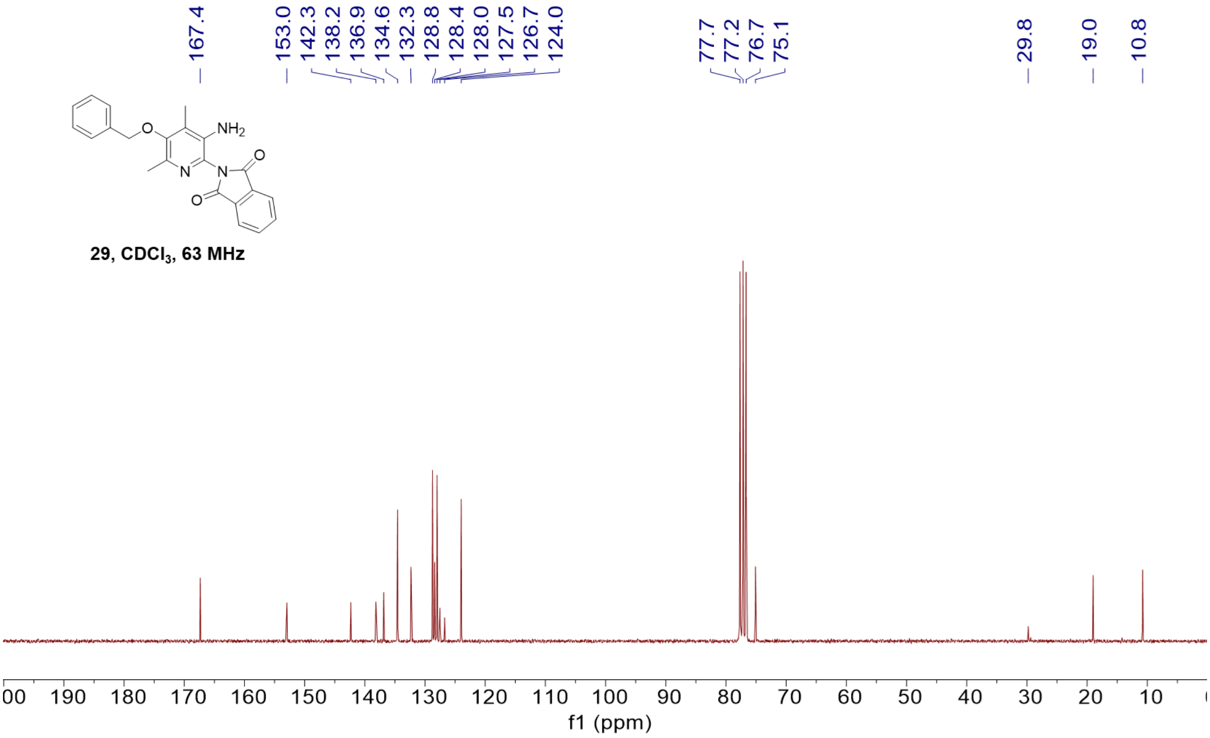

Figure S10. ^{13}C-NMR Spectrum of compound **29**.

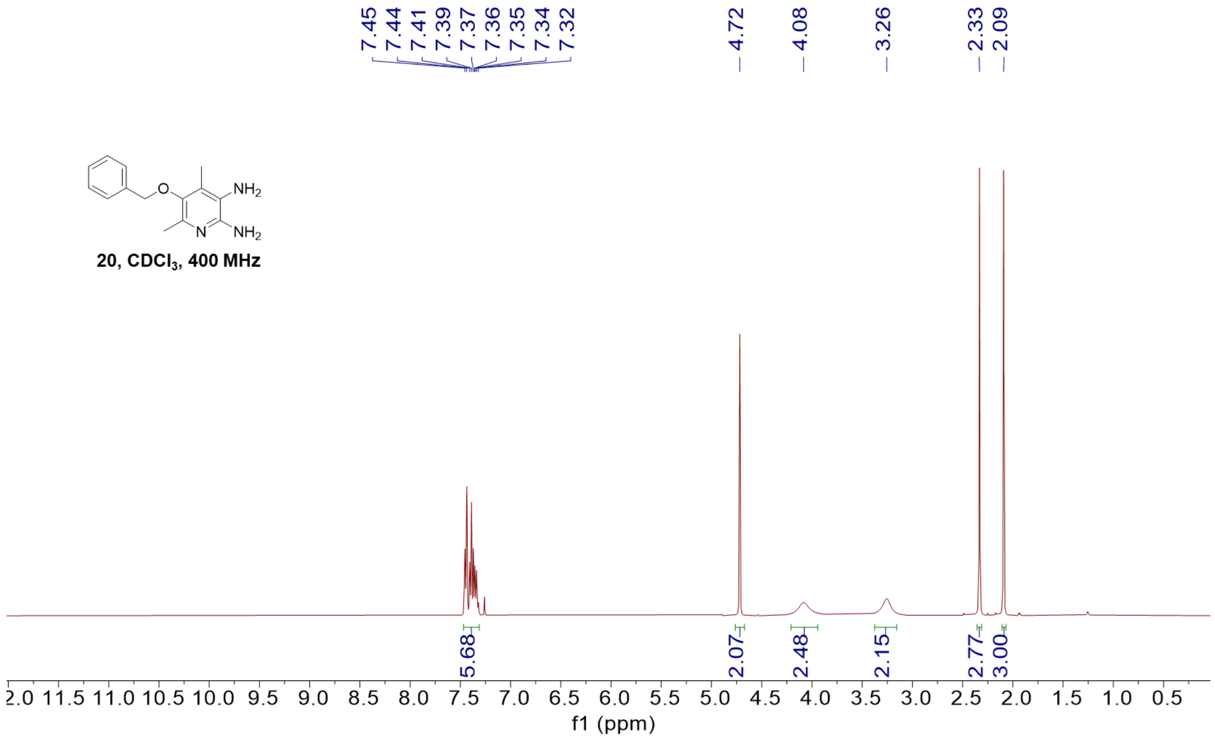

Figure S11. ^{1}H-NMR Spectrum of compound **20**.

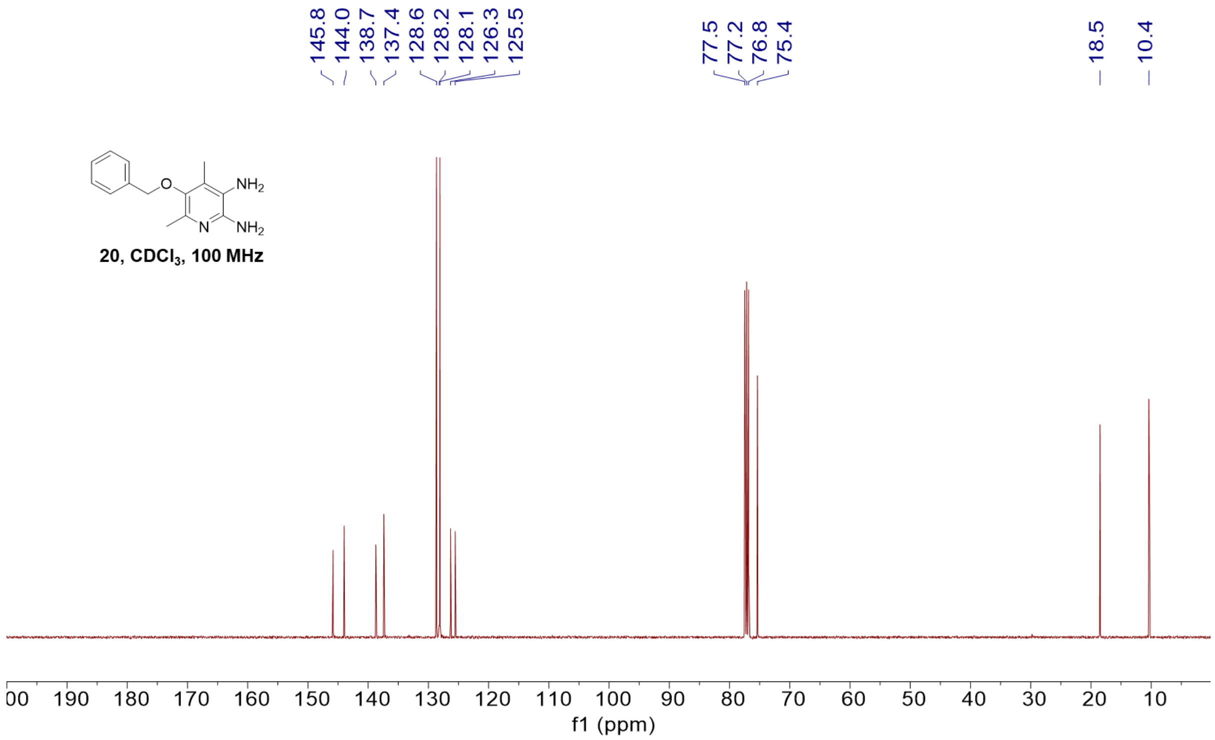

Figure S12. ^{13}C-NMR Spectrum of compound **20**.

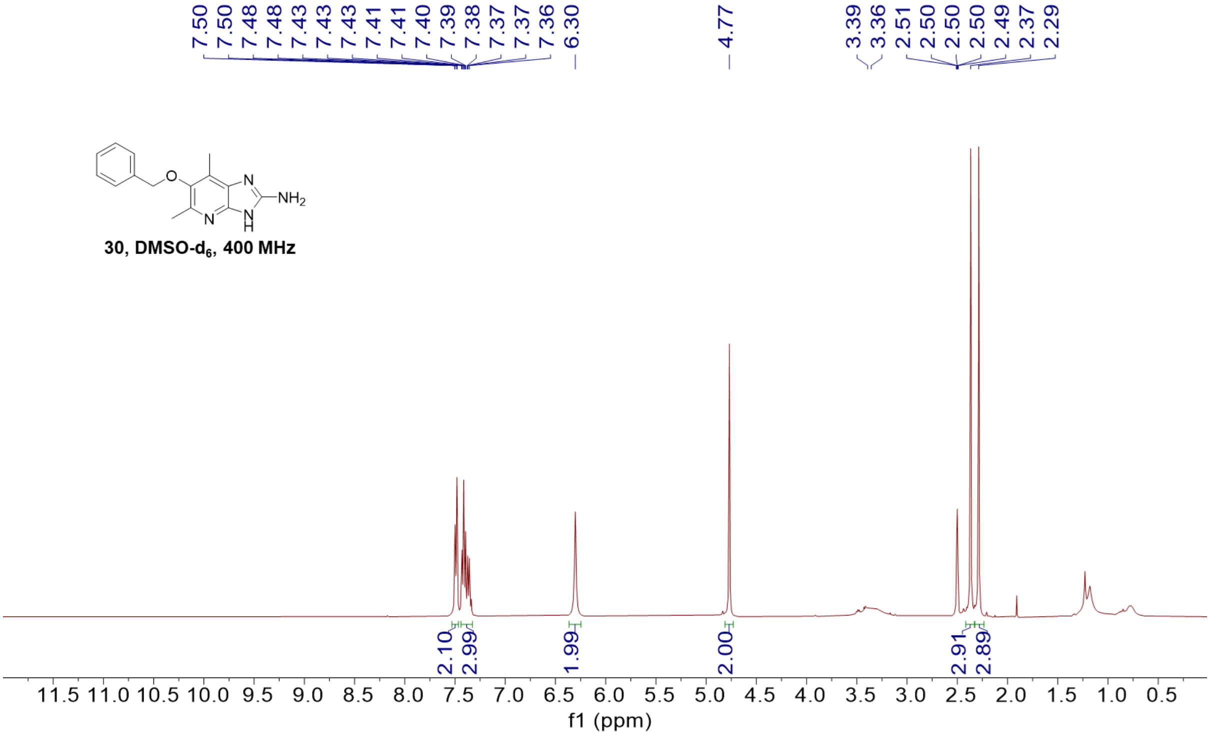

Figure S13. ^{1}H-NMR Spectrum of compound **30**.

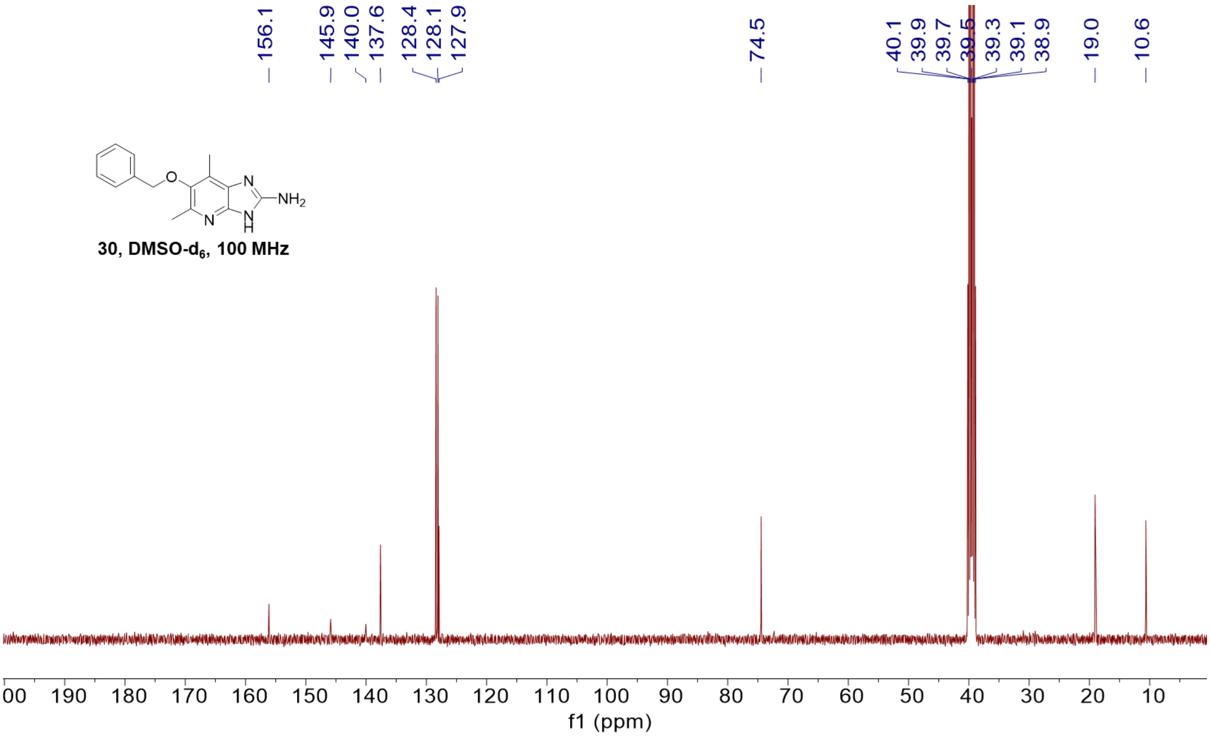

Figure S14. ^{13}C-NMR Spectrum of compound **30**.

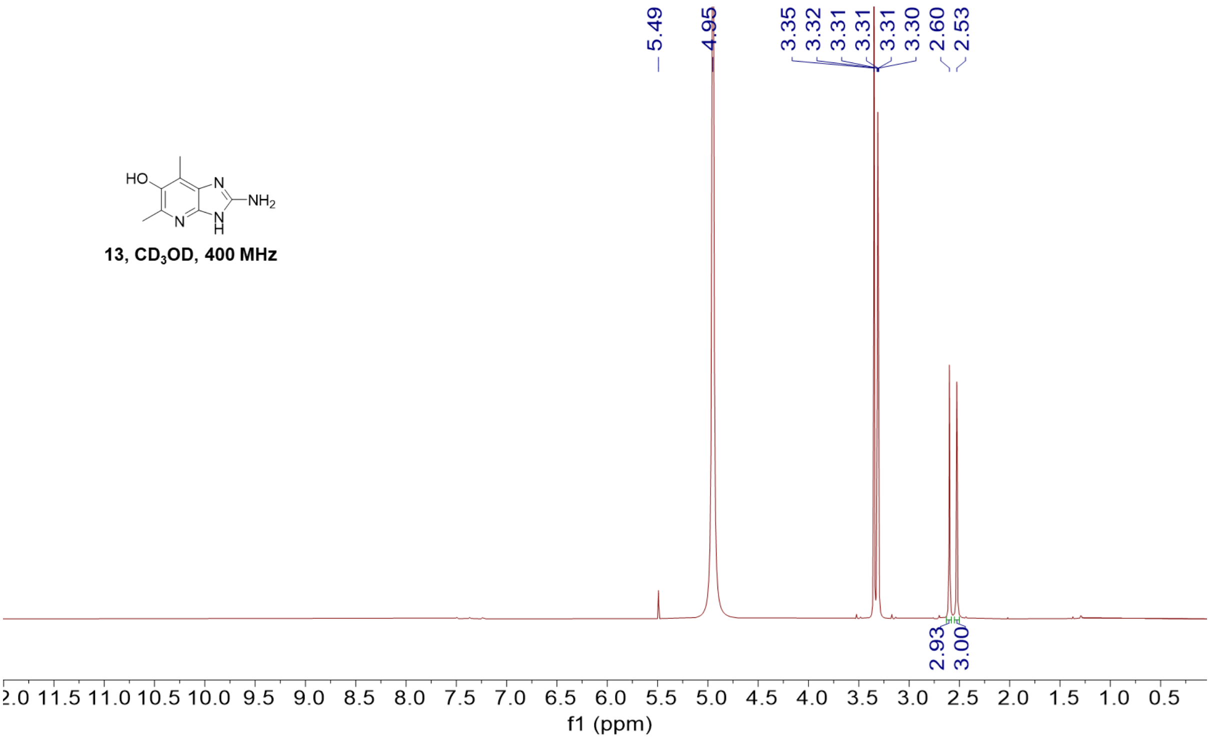

Figure S15. ^{1}H-NMR Spectrum of compound **13**.

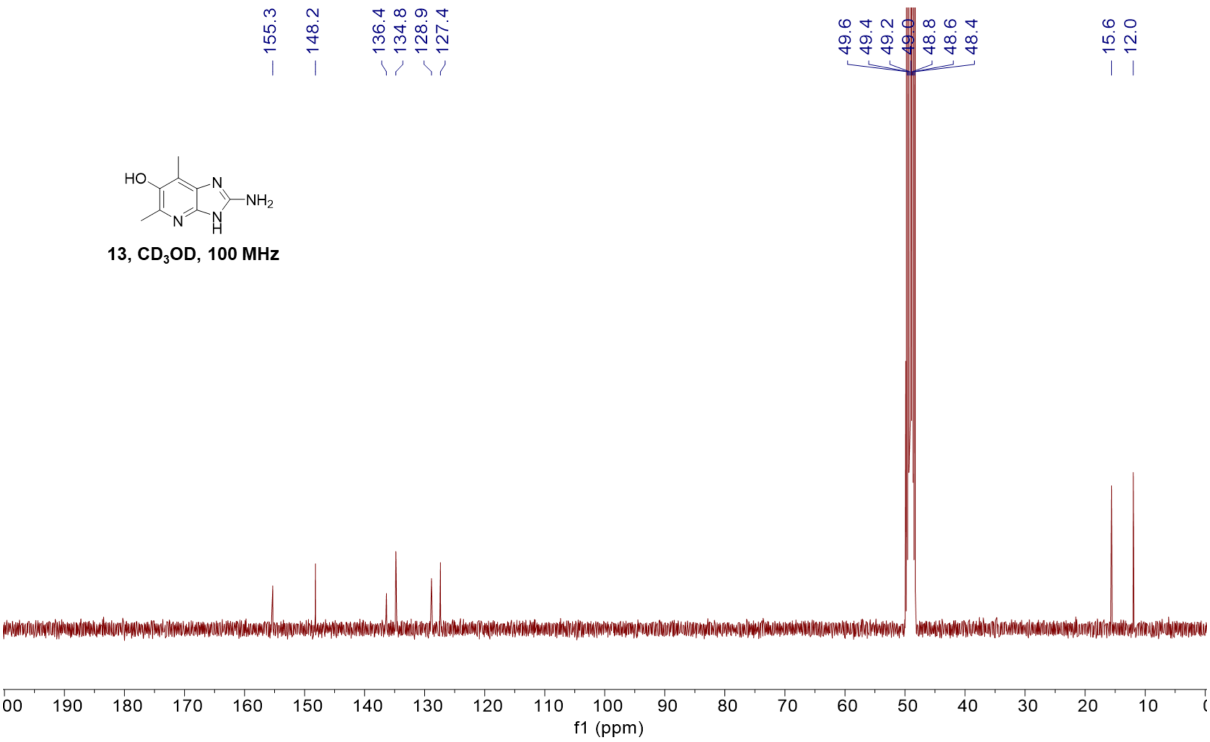

Figure S16. ^{13}C-NMR Spectrum of compound **13**.

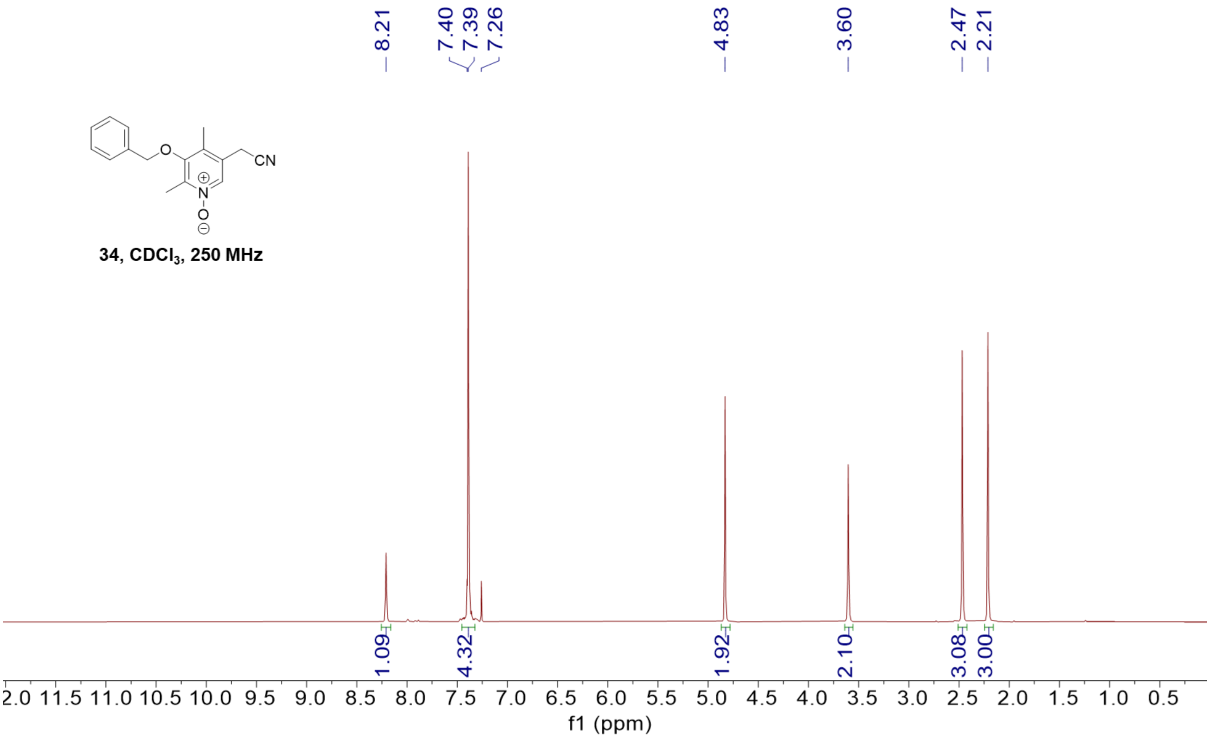

Figure S17. ^{1}H-NMR Spectrum of compound **34**.

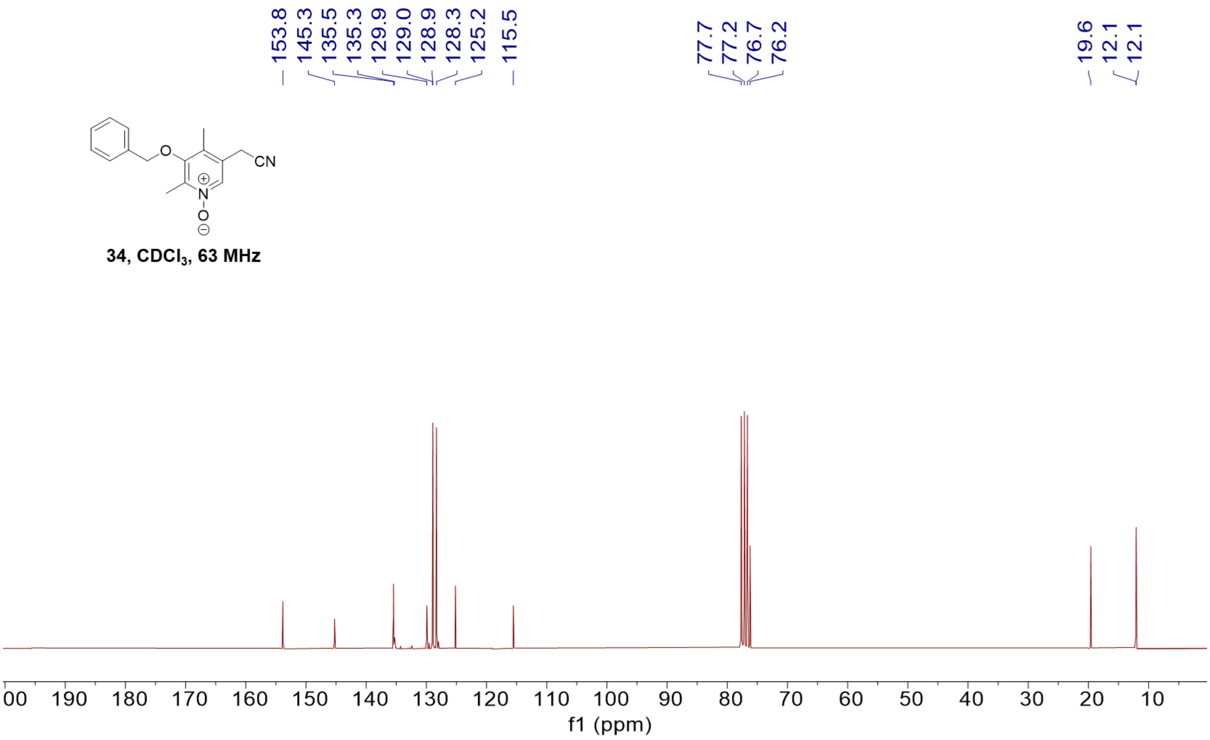

Figure S18. ^{13}C-NMR Spectrum of compound **34**.

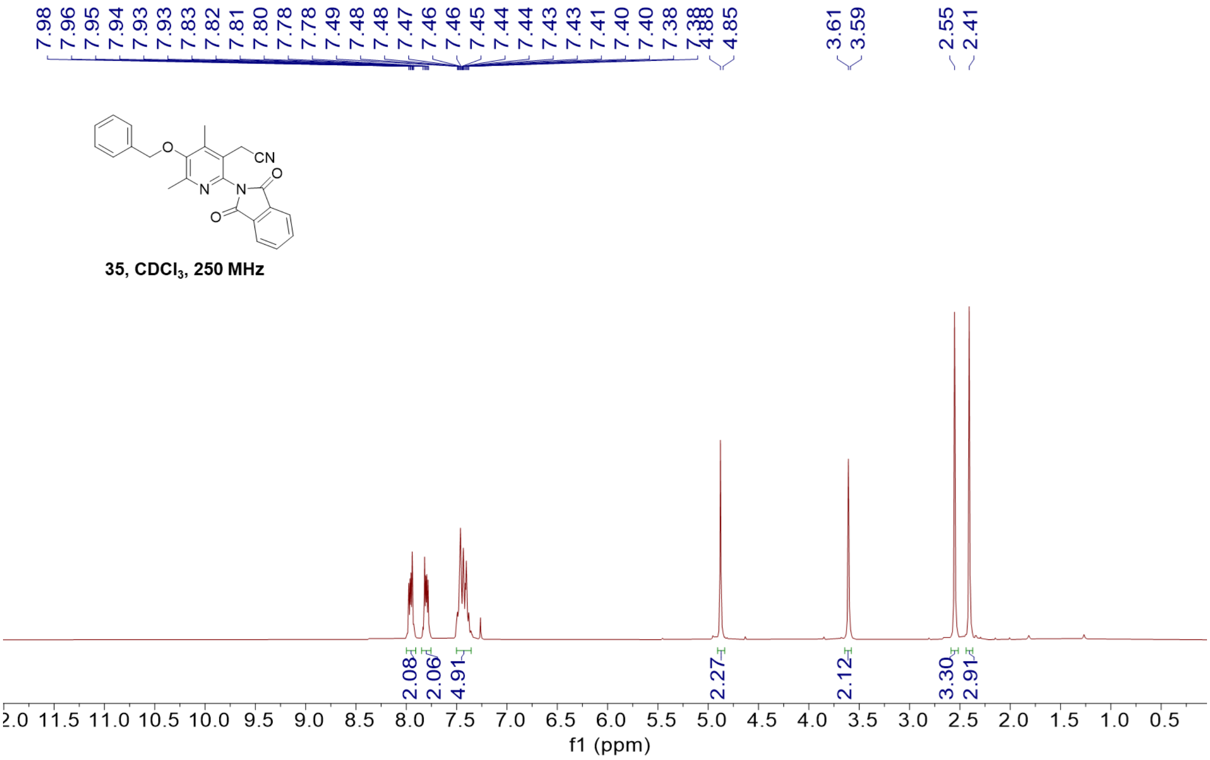

Figure S19. ^{1}H-NMR Spectrum of compound **35**.

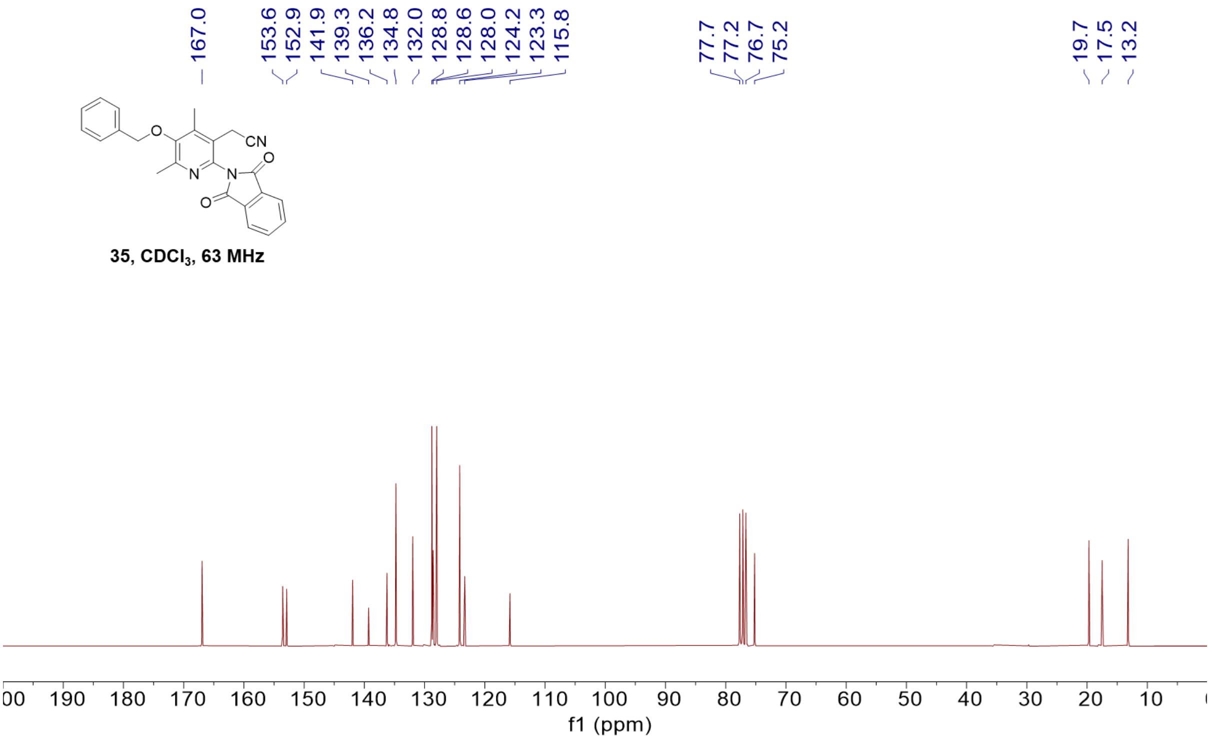

Figure S20. ^{13}C-NMR Spectrum of compound **35**.

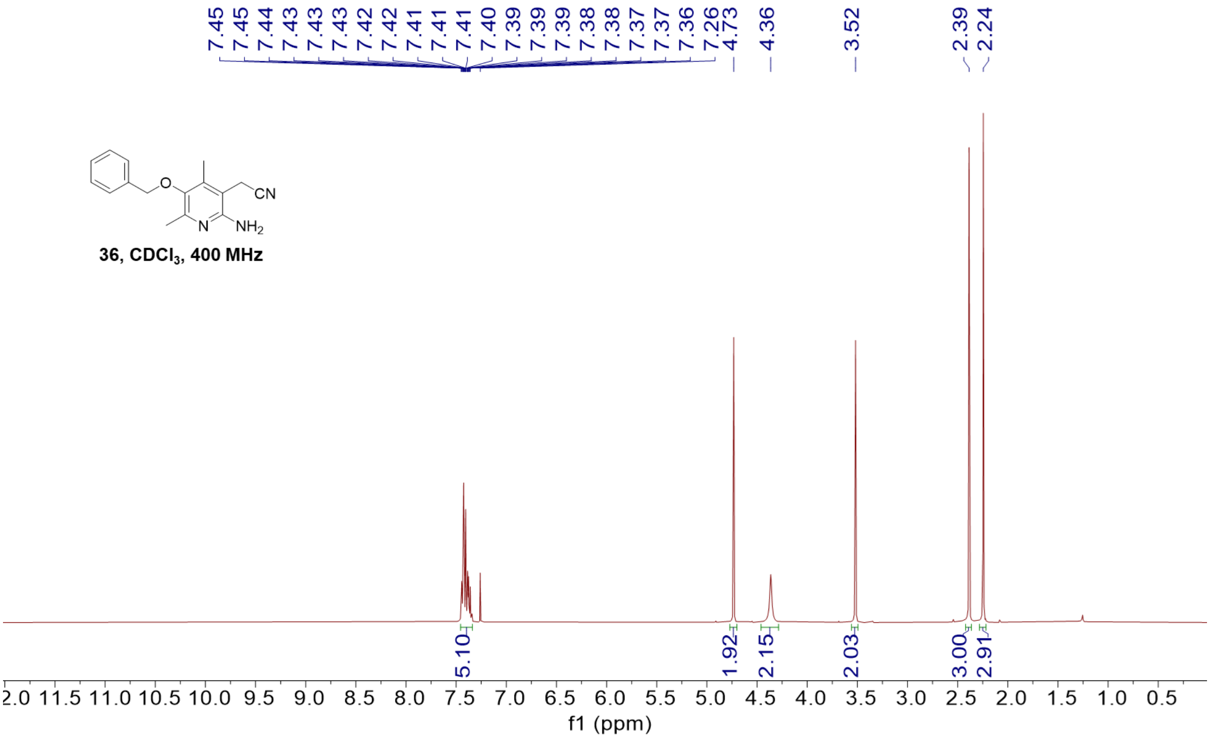

Figure S21. ^{1}H-NMR Spectrum of compound **36**.

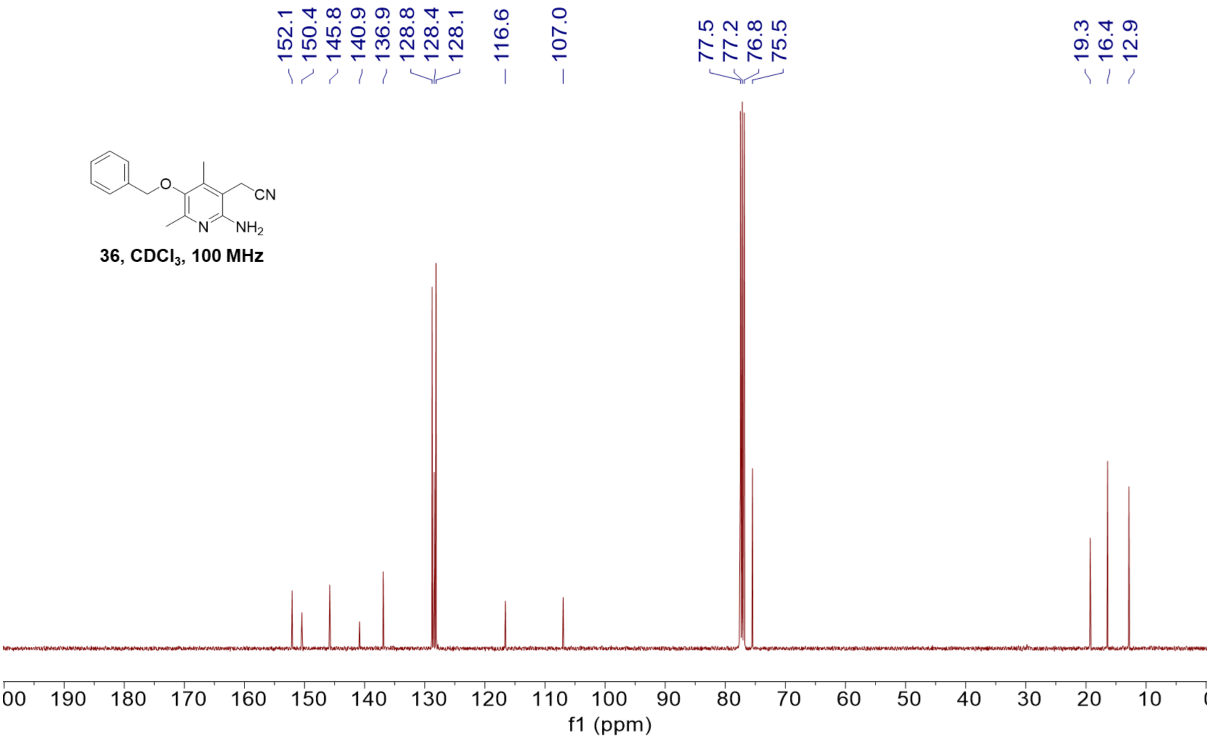

Figure S22. ^{13}C-NMR Spectrum of compound **36**.

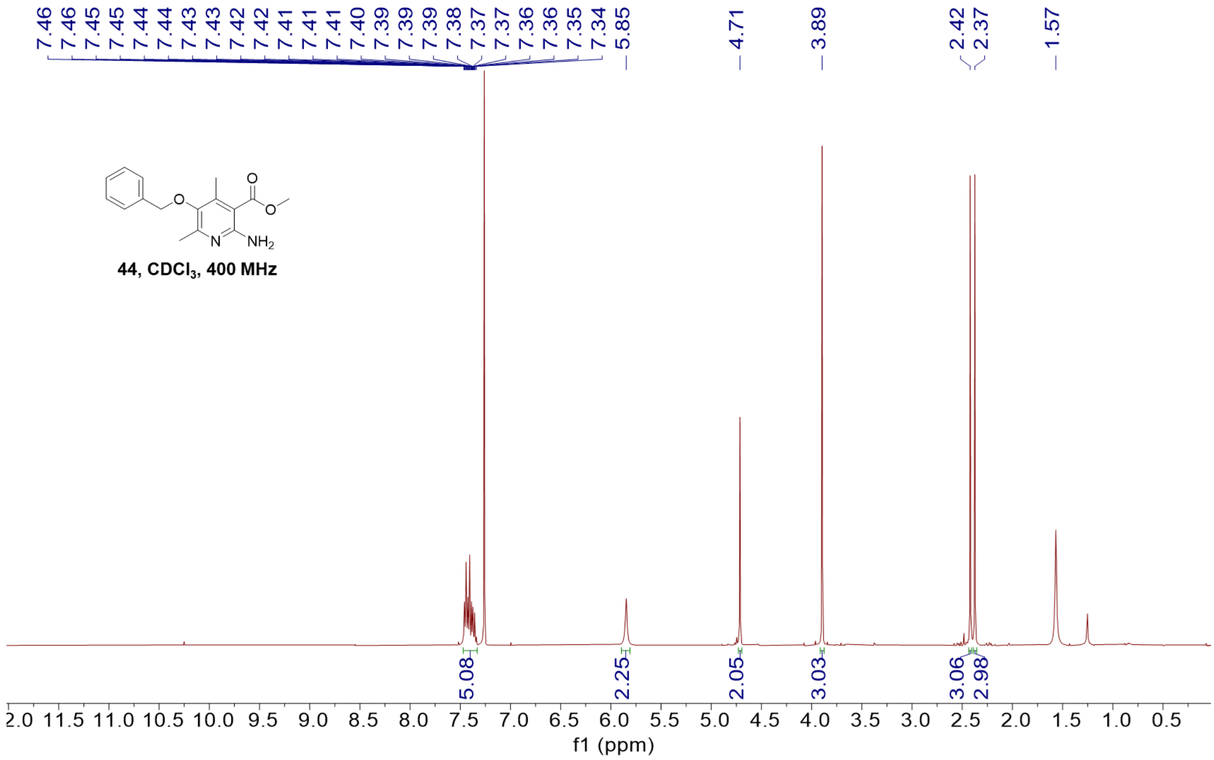

Figure S23. ^{1}H-NMR Spectrum of compound **44**.

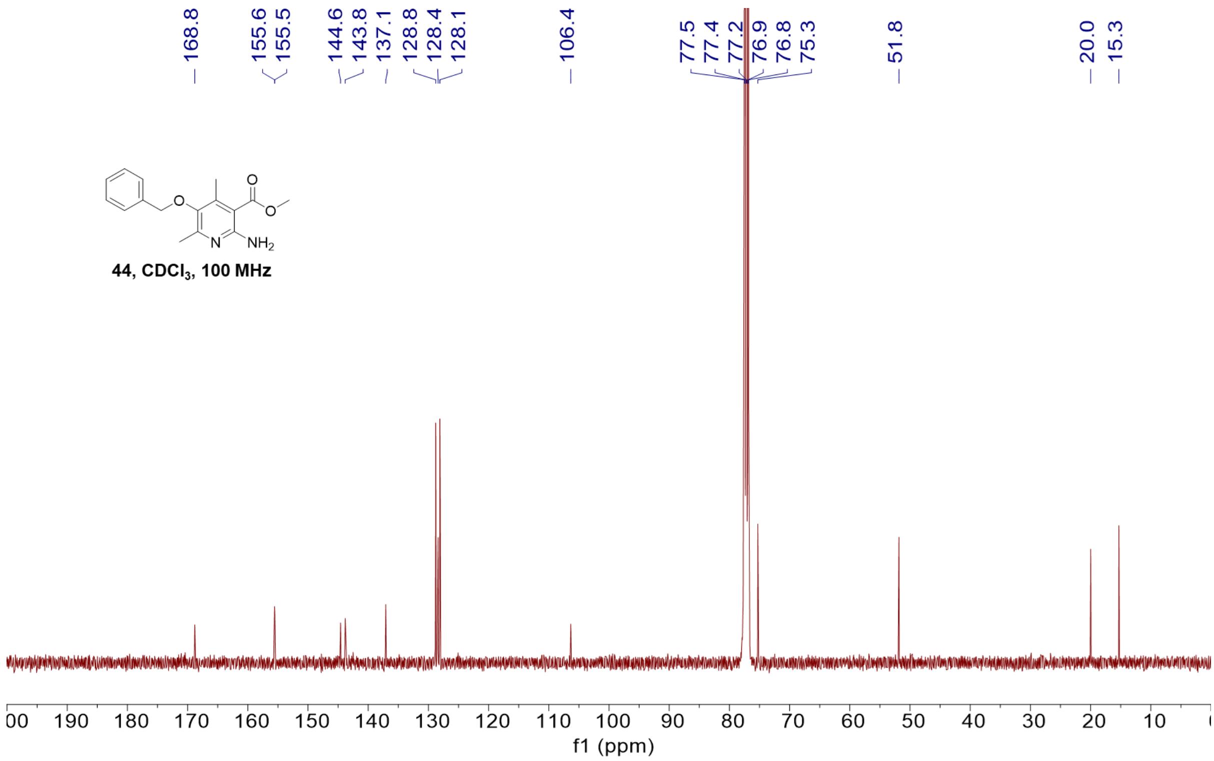

Figure S24. ^{13}C-NMR Spectrum of compound **44**.

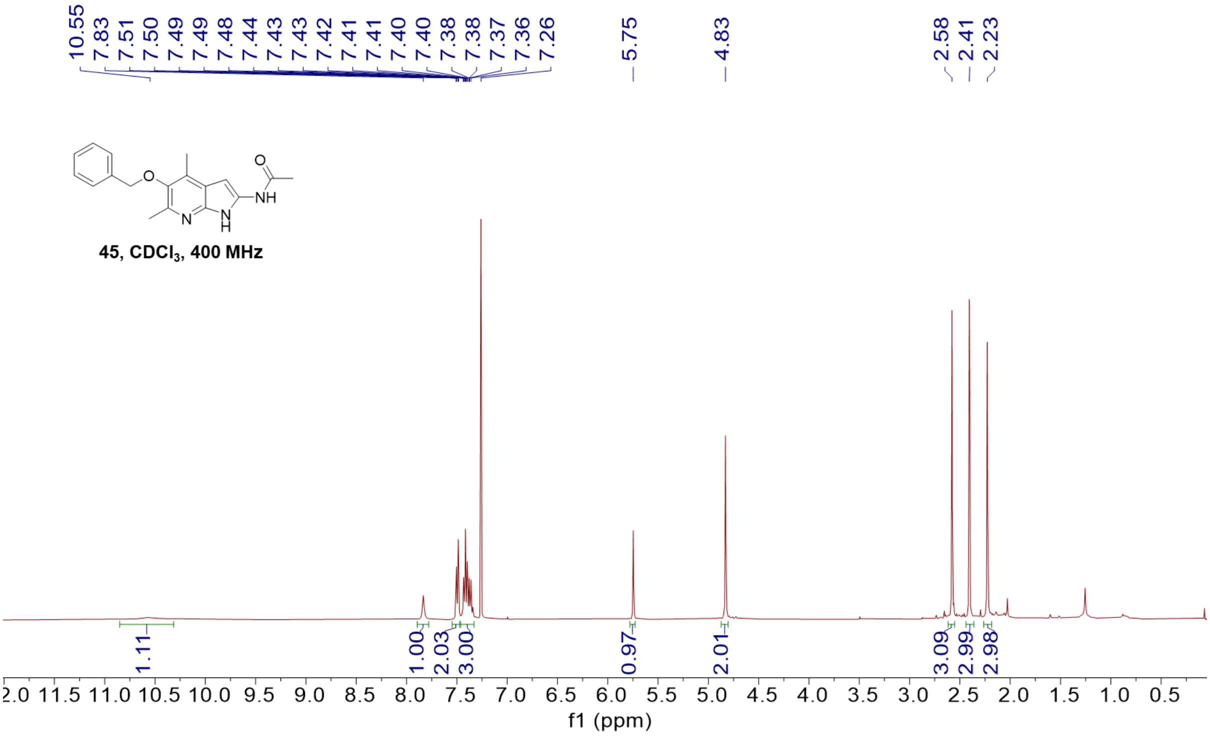

Figure S25. ^{1}H-NMR Spectrum of compound **45**.

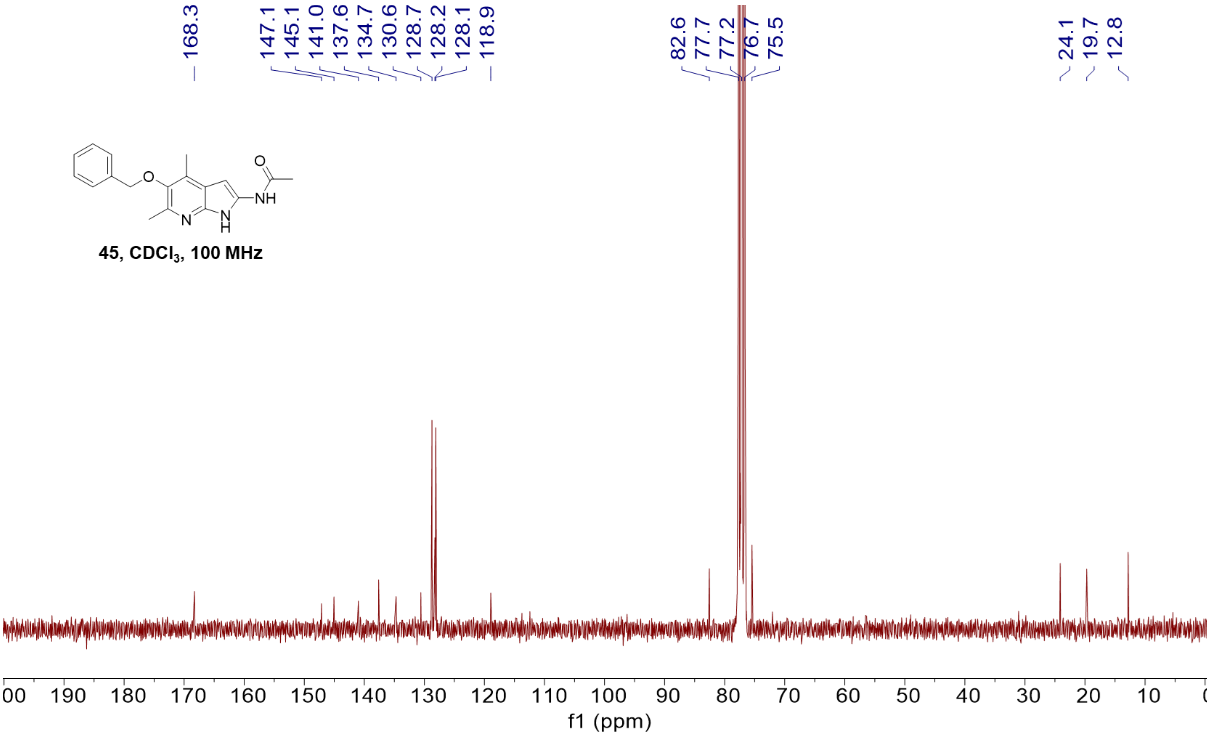

Figure S26. ^{13}C-NMR Spectrum of compound **45**.
